# Over-expression of a γ-tocopherol methyltransferase gene in vitamin E pathway confers PEG-simulated drought tolerance in alfalfa

**DOI:** 10.1186/s12870-020-02424-1

**Published:** 2020-05-19

**Authors:** Jiangtao Ma, Deyun Qiu, Hongwen Gao, Hongyu Wen, Yudi Wu, Yongzhen Pang, Xuemin Wang, Yuchang Qin

**Affiliations:** 1 Institute of Animal Sciences, Chinese Academy of Agricultural Sciences, Beijing, 100193 China; 2 Division of biomedical science and biochemistry, Research School of Biology, The Australian National University, Canberra, ACT 2601 Australia

**Keywords:** Alfalfa, Tocopherol, Abscisic acid (ABA), Stomata, Photosynthesis, RNA-seq, Drought stress

## Abstract

**Background:**

α-Tocopherol is one of the most important vitamin E components present in plant. α-Tocopherol is a potent antioxidant, which can deactivate photoproduced reactive oxygen species (ROS) and prevent lipids from oxidation when plants suffer drought stress. γ-Tocopherol methyltransferase (γ-TMT) catalyzes the formation of α-tocopherol in the tocopherol biosynthetic pathway. Our previous studies showed that over-expression of *γ-TMT* gene can increase the accumulation of α-tocopherol in alfalfa (*Medicago sativa*). However, whether these transgenic plants confer increased drought tolerance and the underlying mechanism are still unknown.

**Results:**

In the present study, we further evaluate transgenic alfalfa lines, and found that over-expression of *MsTMT* led to an increase in α-tocopherol and total tocopherol level in the transgenic lines compared with the control plant. It was revealed that drought tolerance of the transgenic alfalfa was remarkably increased, with alleviated oxidative damage and accumulation of more osmolytic substances. The stomatal development in transgenic plants was significantly inhibited on both sides of leaves, which may be resulted from the repression of *MsSPCHLESS* (*MsSPCH*) gene. The reduced stomatal density of transgenic plants contributes to a lower stomatal conductance and higher water use efficiency (WUE). Moreover, both RNA-seq and qRT-PCR analyses indicate that regulatory mechanism of *MsTMT* in drought involved in both ABA-dependent and ABA-independent pathways.

**Conclusion:**

Our results suggest that *MsTMT* gene plays a positive role in regulating alfalfa response to PEG-simulated drought stress, which might involve complex mechanisms, including ROS scavenging system, stomatal development and multiple phytohormone signaling pathways. This study will broaden our view on the function of *γ-TMT* gene and provide new strategy for genetic engineering in alfalfa breeding.

## Background

As sessile organism, plants are always exposed to a range of abiotic stresses such as drought, salt, adverse temperature and heavy metals. Among these stresses, drought is one of the greatest natural constraints to agricultural production, which can result in more than 10% decrease in crop production [1]. Biochemical studies revealed that drought stress is associated with an increase in photosynthesis-derived reactive oxygen species (ROS), which includes superoxide anion, hydroxyl radical and hydrogen peroxide [2–4]. Chloroplast thylakoids are the primary site in which ROS are photo-produced from successive reduction of molecular oxygen [2, 3]. ROS are relatively reactive and potentially harmful to plants due to unspecific oxidation of proteins and membrane lipids. To eliminate these damage, plants have evolved protective mechanisms to maintain delicate equilibrium between ROS production and scavenging [2, 4–6].

Drought tolerance and avoidance may be also achieved by increasing water use efficiency (WUE), which is controlled by stomata that modulate transpiration flux, water use and CO_2_ uptake on leaf surface [7–10]. Based on previous studies, it is suggested that reducing stomatal density is possibly an effective way to improve WUE and drought tolerance. Several genes have been found to be involved in the regulation of stomatal density. Overexpression of *EPF1*, a gene encoding a cysteine-rich secreted peptide, led to an increase in WUE by reducing stomatal density and improved drought tolerance in transgenic *Arabidopsis*, poplar and barley [11–13]. *ANGUSTIFOLIA3* (*AN3*) encodes a homolog of the human transcription coactivator SYT, which can regulate WUE and drought tolerance by reducing stomatal density [14]. When a maize B3 domain factor gene, *ZmBDF*, was ectopically expressed in *Arabidopsis*, the transgenic plants conferred drought and salt tolerance and displayed reduced stomatal density and gas exchange under drought stress [15].

Tocopherols, the collective term of four structurally similar complex (α-, β-, γ- and δ-), are strong antioxidants that could prevent lipids from oxidation by providing hydrogen atom of the chromanol head to lipid peroxy radical oxygen [16–18]. Tocopherols are also highly effective quenchers or chemical scavengers of all forms of activated oxygen. Among them, α-tocopherol is considered to have the highest biological activity in animals. In plants, α-tocopherol usually accounts for more than 90% of the foliar vitamin E content [19–22].

**Fig. 1:**
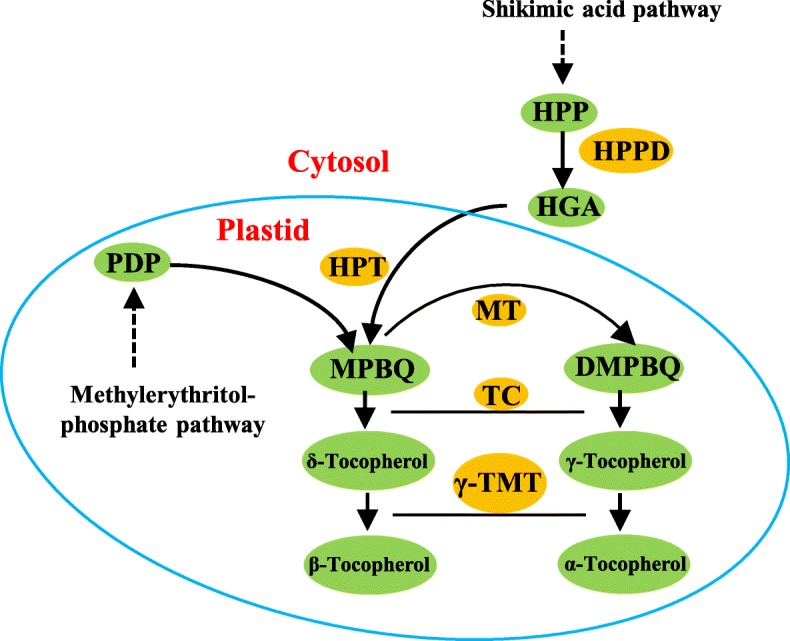
The biosynthetic pathway of tocopherols in plants. G3P: glyceraldehyde 3-phosphate; PA: pyruvic acid; HPP: hydroxyphenylpyruvate; HPPD: HPP dioxygenase; PDP: phytyl diphosphate; HGA: homogentisic acid; HPT: HGA phytyltransferase; MPBQ: 2-methyl-6-phytyl-1,4-benzoquinol; DMPBQ: 2,3-dimethyl-6-phytyl-1,4-benzoquinol; MT: MPBQ methyltransferase; TC: tocopherol cyclase; γ-TMT: γ-tocopherol methyltransferase

The enzyme γ-tocopherol methyltransferase (γ-TMT) catalyzes the methylation of γ- and δ-tocopherol to α- and β-tocopherol, respectively, and it is the last key enzyme in tocopherol biosynthesis pathway (Fig. 1). Loss of function mutation in *γ-TMT* led to decrease in the rosette dry weight by 37% in *Arabidopsis*. Meanwhile, the *vte4* mutant of *Arabidopsis* also scored a lower soil water potential than the wild-type plants [23], suggesting that tocopherol content may be associated with WUE and drought tolerance. Based on these studies, we proposed that increasing α-tocopherol content by over-expressing *γ-TMT* could be an ideal strategy to improve plant drought tolerance.

Alfalfa is an important perennial legume crop grown worldwide with high nutritional value. Improving WUE and drought tolerance are key targets in alfalfa breeding in the context of global climate change. To obtain a deeper insight into the mechanisms of *γ-TMT* gene in water use and drought stress, we over-expressed the *M. sativa TMT* gene (*MsTMT*) in alfalfa and performed PEG-simulated drought stress in these transgenic plants. In the transgenic alfalfa constitutively expressing *MsTMT*, both α-tocopherol and total tocopherol content were increased. Transgenic alfalfa showed reduced stomatal density on both sides of leaves, and the expression level of *M. sativa* SPEECHLESS (*MsSPCH*) gene was suppressed, which dramatically decreased transpiration rate and contributed to increased transpiration efficiency. Overexpression of *MsTMT* also conferred increased drought tolerance that involved water holding capacity, antioxidant activity and osmotic adjustment in leaves. Our results suggest that tocopherol was associated with plant response to drought stress, which involves WUE, multiple phytohormone signaling and ROS scavenging system in the transgenic alfalfa plants. This study reveals that over-expression of *γ-TMT* could affect stomatal development and response to drought stress, which will be useful for engineering alfalfa with improved vitamin E content, WUE and drought tolerance.

## Results

### Ectopic expression of *MsTMT* increases α-tocopherol content and drought tolerance in transgenic alfalfa

**Fig. 2:**
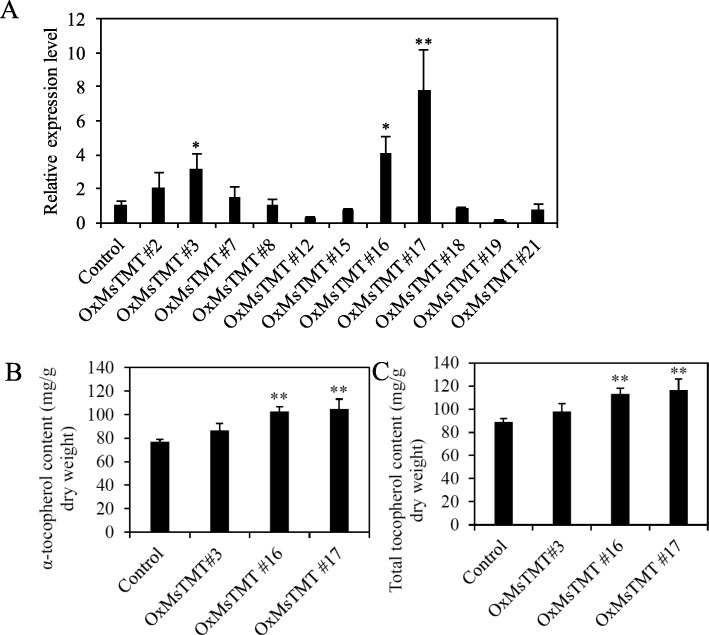
Molecular analysis of the transgenic alfalfa lines over-expressing *MsTMT*. **a**. Analysis of *MsTMT* gene expression level in transgenic plants by qRT-PCR. **b, c**. α-Tocopherol and total tocopherols content of transgenic lines and control plants in mature alfalfa leaves. Values are the mean ± SEM. Significant differences: *P ≤ 0.05, **P ≤ 0.01, ***P ≤ 0.001

In a previous study, we have generated a number of transgenic alfalfa lines that over-expressing the *MsTMT* gene (Fig. S1; Fig. S2) [24]. To re-evaluate these transgenic lines, the expression levels of *MsTMT* gene in eleven transgenic alfalfa lines (after propagation by cutting) were further confirmed by using qRT-PCR (Fig. 2a). Of these lines, three new representative ones, Nos. 3, 16 and 17, with a relatively high expression level, were selected for the measurement of tocopherol content. It revealed that α-tocopherol and total tocopherol in transgenic line No. 17 with the highest *MsTMT* gene expression level were respectively increased by 36 and 31% in comparison with the vector control line (Fig. 2b-c, Fig. S3).

**Fig. 3:**
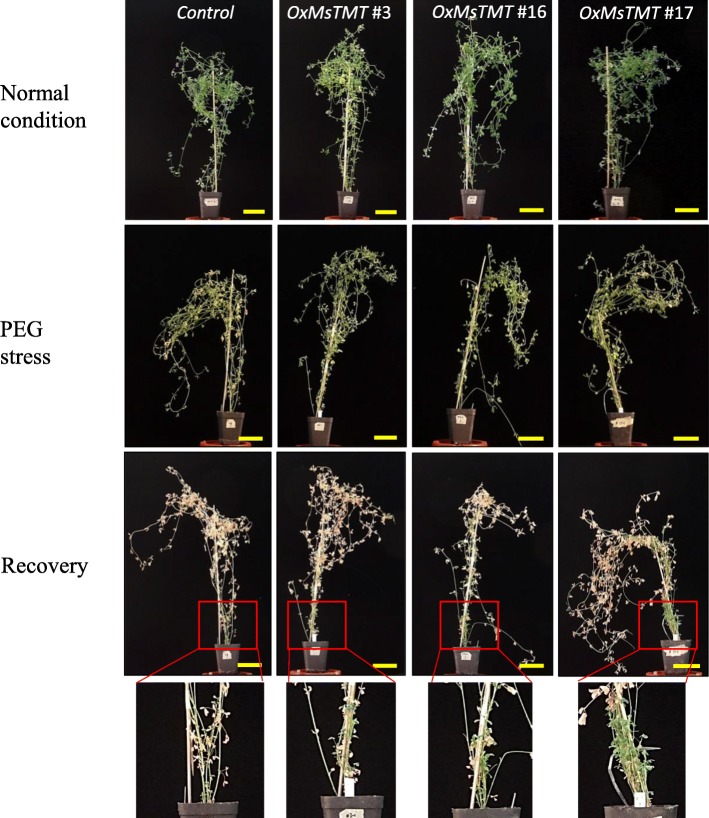
Plant phenotype under the normal condition (upper panel), 20% PEG stress for 7 days (middle panel) and recovered after re-watering for 10 days (lower panel). The red square indicate new branches generated from the root crown of plants. Scale bar = 5 cm

To further investigate potential drought tolerance of these transgenic lines, 4-week-old transgenic lines were subjected to a simulated drought stress in 20% PEG solution together with control plants. After a 7-day treatment, both the control and transgenic alfalfa plants showed retarded growth and chlorotic symptoms, but the damage seemed to be worse in the control than in the transgenic lines (Fig. 3). After rehydration for 5 days, leaves in the control plants were all withered and turned yellow (Fig. 3). However, leaves of the transgenic alfalfa recovered quickly, and new branches were regenerated from the root crown, particularly in line No. 17 (Fig. 3).

**Fig. 4:**
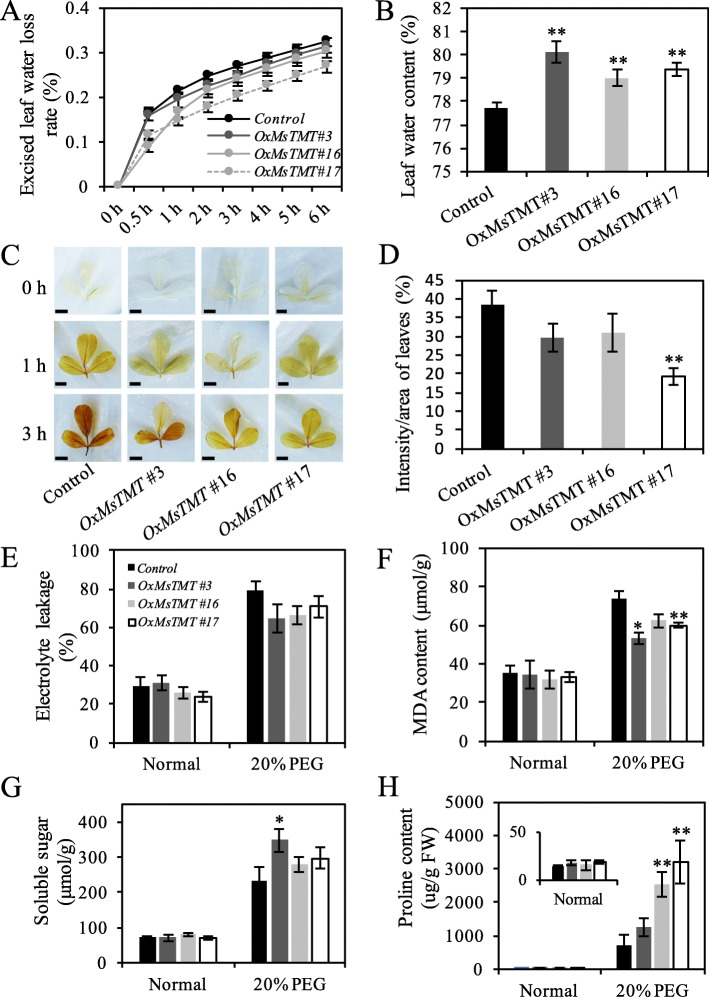
Comparison of physiological indexes of transgenic lines and control plants under stress condition. **a**. Water loss rates were determined at 0, 0.5, 1, 2, 3, 4, 5 and 6 h. **b**. Water content of leaves. **c-d**. Histochemical detection of H_2_O_2_ in leaves of control and transgenic lines after water loss at indicated times. Scale bar = 0.5 cm. Quantification of DAB staining after 72 h PEG treatment with Image j. **e-h**. Relative electrolyte leakage, MDA content, and soluble sugar and proline content of mature leaves under stress. Each value indicates the mean ± SEM. Significant differences: *P ≤ 0.05, **P ≤ 0.01

Leaf water content and loss rate are common evaluating parameters of plant drought resistance ability. Detection of the water content loss from the detached leaves revealed that all transgenic lines showed much lower water loss than the control plants after the first 0.5 h (Fig. 4a). In particular, water content loss was the lowest in line No. 17, which was consistent with the highest expression level of *MsTMT* (Fig. 4a). Meanwhile, water content of transgenic lines was also significantly higher than that of the control alfalfa under normal growth condition (Fig. 4b).

To determine the oxidative damage induced by stress in leaves, the dehydrated leaves were collected and treated with the diaminobenzidine (DAB) staining. Significantly increased DAB staining was found in the control leaves, but a lighter staining in transgenic leaves, especially for line No. 17 (Fig. 4c, d), suggesting that ROS levels were lower in the transgenic plants than in the control plants under drought treatment. To determine whether cell membrane was damaged under drought stress, electrolyte leakage (EL) and malondialdehyde (MDA) content for cell membrane integrity was determined. It showed that both the control and transgenic plants had similar EL and MDA content under normal condition, but MDA content for the transgenic lines were much lower than the control plants under drought stress, while only slight decreases were found in EL for transgenic lines (Fig. 4e, f).

Moreover, transgenic lines exhibited higher soluble sugar under PEG stress though only line No. 3 showed a significant increase by 49%, but no significant difference were found among transgenic lines (Fig. 4g). Proline concentrations of control and transgenic plants were at similar level under normal condition, but those in transgenic lines 16 and 17 were dramatically increased when suffering from PEG stress compared with those in control plants (Fig. 4h). Proline contents in these two transgenic lines were increased by 2.6 and 3.5 times, respectively (Fig. 4h).

### Gas exchange and WUE were improved in transgenic alfalfa leaves

**Fig. 5:**
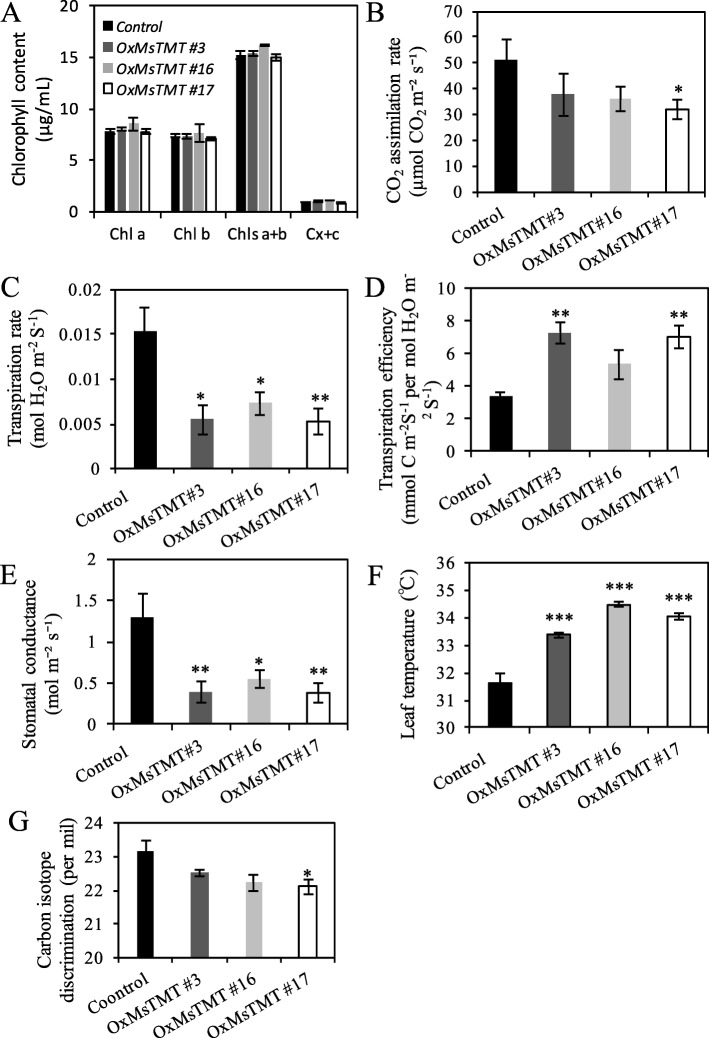
Overexpression of the *MsTMT* gene improved water use efficiency in alfalfa. **a**. Relative chlorophyll content of mature leaflets. **b-e**. CO_2_ assimilation rate, transpiration rate, transpiration efficiency and stomatal conductance were estimated from gas exchange measurements on mature leaflets using the LI-COR 6800 system. **f**. Temperature of mature leaflets was obtained with the chlorophyll analyzer. The first fully expanded leaf from top of branch of each plant was selected to analysis in those measurements. **g**. Analysis of leaf carbon isotope composition (δ) of transgenic alfalfa. The first fully expanded leaf from top of branch was collected for analysis. Values are the mean ± SEM. Significant differences: *P ≤ 0.05, **P ≤ 0.01, ***P ≤ 0.001

Previous study showed that overexpressing *γ-TMT* gene enhances tolerance of plants under various abiotic stresses by affecting photosynthetic performance [25], therefore, leaf chlorophyll, as one of the essential pigments for photosynthesis, was examined in both the transgenic and control plants. However, no significant difference in the contents of chlorophyll a and b were found between them (Fig. 5a).

Further analysis revealed that the transgenic plants maintained lower carbon assimilation rate, especially for line No. 17, and much lower transpiration rate (Fig. 5b-c, Fig. S4) under normal growth condition, which ultimately resulted in a 2.1- to 2.0-fold increase in transpiration efficiency in line Nos. 3 and 17 (Fig. 5d). The stomatal conductance was also lower in transgenic lines than in the control (Fig. 5e). Thus, transgenic plants maintained a significantly higher leaf temperature than the control plants, which possibly contributed by lowered transpiration rate and stomatal conductance (Fig. 5f).

High WUE can help plants to maintain high survival rate under water limitation. Hence, WUE was commonly used as an indicator in drought tolerance assay. To confirm WUE calculated from photosynthesis and transpiration rate (Fig. 5D), we determined carbon isotope composition and calculated the △ value. The transgenic lines displayed a lower △ value than the control plants (Fig. 5g). The changes of △ were 0.66–1.07 per mil on average in comparison with the control, indicating an approximate 17–28% increase in WUE, which is consistent with the result in Fig. 5d.

### Stomatal density was reduced in the transgenic alfalfa leaf

**Fig. 6:**
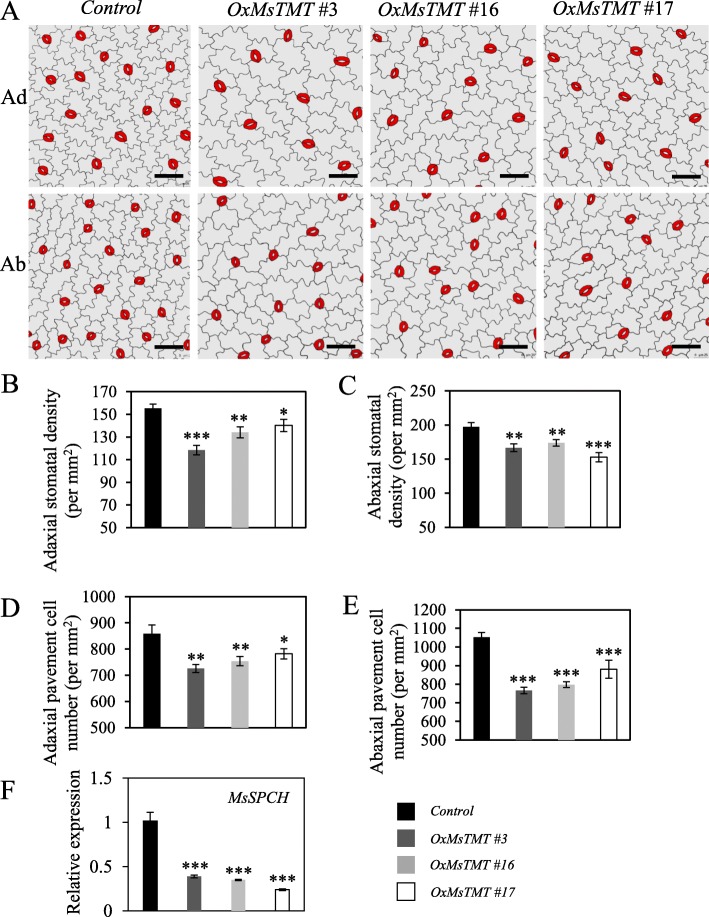
The effects of *MsTMT* on stomatal morphology and development. A. Epidermal tracing from alfalfa mature leaflet overexpressing *MsTMT*. Scale bar = 50 μm. B and C. Overexpression of *MsTMT* led to a decrease in stomatal density on adaxial and abaxial sides. D and E. Pavement cell number was reduced on both leaflet sides of transgenic lines. Values are the mean ± SEM. Significant differences: *P ≤ 0.05, **P ≤ 0.01, ***P ≤ 0.001

We speculated that the changes in gas exchange in transgenic lines might be affected by stomatal density or opening. To validate this hypothesis, epidermis morphology was investigated, and it showed that the transgenic lines had fewer stomata than the control plants on both sides of the leaflets (Fig. 6a). Meanwhile, no significant difference in stomatal opening was noticed between control and transgenic lines during the observation. Leaf adaxial stomatal density in transgenic plants decreased by 36.6% for line No. 3 and the abaxial side by 22.6% for line No. 17 (Fig. 6b, c), suggesting that the increase in WUE of the transgenic lines could be due to the reduction in stomatal density.

Pavement cells were larger in leaflets of the transgenic lines, which resulted in a lower pavement cell density on both sides of leaflets than the control (Fig. 6d, e).

The decrease in stomatal density led us to suspect that cell division might be restrained during stomatal development in transgenic lines. Therefore, we measured the transcript level of a *SPEECHLESS* (*SPCH*) gene, which is necessary for the initial asymmetric cell division of the meristemoid mother cell (MMC) in the establishment of stomatal-lineage ground cells (SLGCs) [26, 27]. qRT-PCR analysis showed the expression of the *MsSPCH* homolog was extremely repressed by 2.6-, 2.9- and 4.2-fold in the transgenic line Nos. 3, 16 and 17 (Fig. 6f), which should contribute to the decrease in their stomatal density. These results suggested that stomatal and pavement cell development including proliferation and expansion were all affected in the transgenic alfalfa plants.

### The expression levels of genes involved in drought tolerance and ABA biosynthesis and signaling were affected in the transgenic alfalfa

**Fig. 7:**
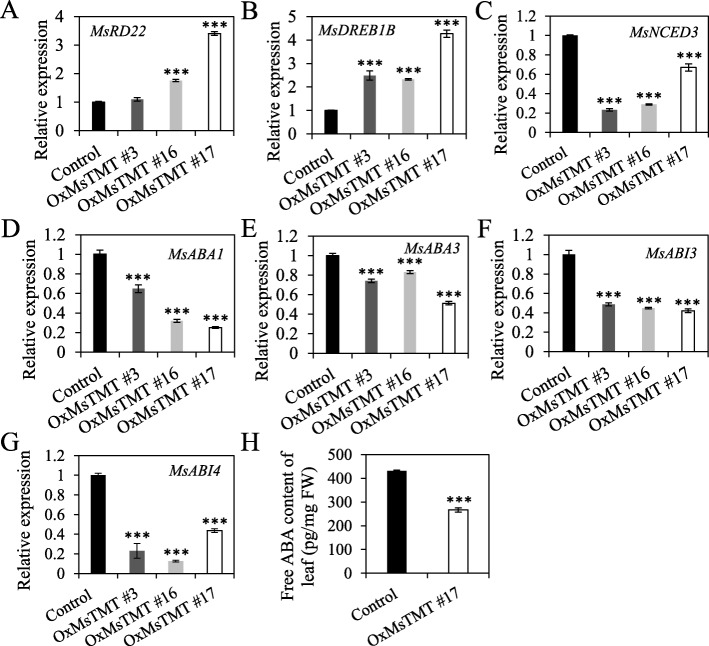
Relative expression of drought stress-related genes and ABA content in transgenic and control plants. *MsActin* (EU664318) and *MsADF* (JZ818469) were used as the reference genes to normalize the gene expression and showed similar results. The 2^-ΔΔ*CT*^_T_ method was used for qRT-PCR analysis with three biological replicates. Values are the mean ± SEM. Significant differences: *P ≤ 0.05, **P ≤ 0.01, ***P ≤ 0.001

Abscisic acid (ABA) plays important roles in regulating plant responses and tolerance to environmental stress [28, 29]. To examine whether the drought stress response in the transgenic lines relies on ABA, the transcript levels of ABA-related genes, including ABA response (*RD22*, *DREB1B*), ABA biosynthesis (*ABA1*, *ABA3* and *NCED3*), and ABA signaling (*ABI3, 4*), were analyzed by qRT-PCR. It showed that the expression levels of *RD22* and *DREB1B* were increased significantly (Fig. 7a, b), whereas the expression levels of genes in ABA biosynthesis and ABA signaling pathway were repressed in the transgenic alfalfa in comparison with the control (Fig. 7c-g). These results indicated that the inhibitory effect on ABA biosynthesis results in the decrease of ABA content. Further quantification revealed 40% reduction in ABA content in leaves of the transgenic lines No. 17 (Fig. 7h, Fig. S5). These results together suggested that both ABA synthesis and signaling pathway were repressed in the transgenic lines.

### Global transcript analysis of gene changes in the transgenic lines by RNA-Seq

To obtain further insight into the molecular mechanism of *MsTMT* involved in drought response, RNA-seq-based transcriptome analysis was performed with leaves of the control and transgenic lines. A total of 288 genes were differentially expressed between the control and transgenic alfalfa (Table S1). Of these, 112 (38.9%) genes were down-regulated, whereas 176 (61.1%) genes were up regulated in the transgenic alfalfa.

Gene ontology (GO) analysis showed that these DEGs were assigned to 38 functional groups that were clustered into three main categories “biological process,” “cellular component” and “molecular function” (Fig. S6A). Among these functional groups, “metabolic process,” “membrane” and “catalytic activity” were the most abundant GO terms in each of the categories, respectively. These DEGs were annotated to the reference pathways and were assigned to five specific pathways. The pathway with the most representation was “carbohydrate metabolism” cluster, followed by “signal transduction,” “biosynthesis of other secondary metabolites” and “environmental adaptation” (Fig. S6B). Further analysis suggested these DEGs were significantly enriched in twenty metabolic pathways in transgenic plants, including “photosynthesis-antenna proteins,” “betalain biosynthesis” and “cAMP signaling pathway” that are critical for plant response and adaptation to adverse environmental conditions (Fig. S6C).

**Fig. 8:**
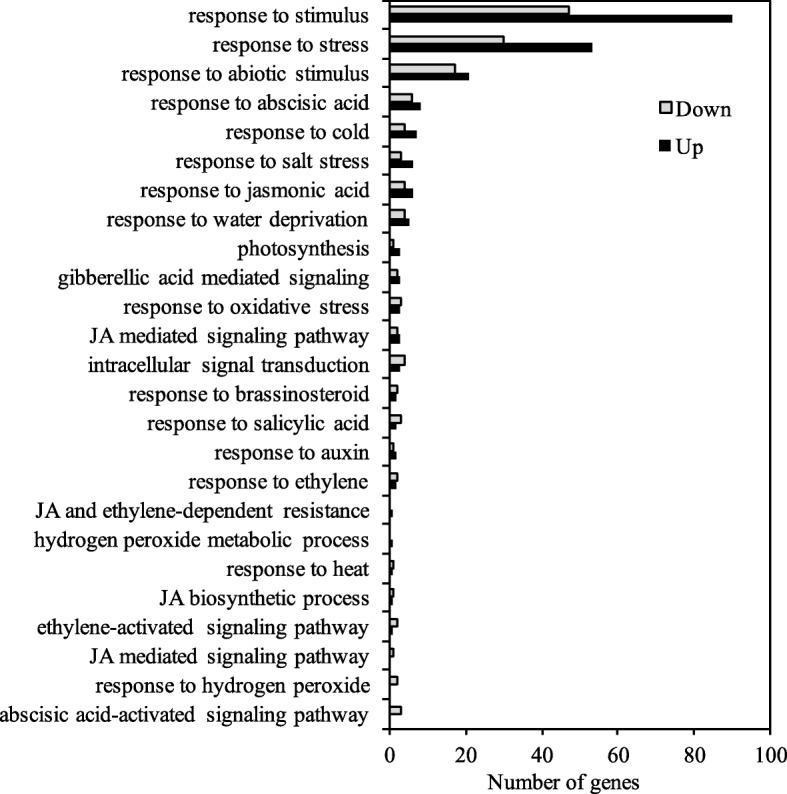
Classification of differentially expressed genes (DEGs) involved in biological processes between control and transgenic plants by GO analysis

Notably, many DEGs were involved in abiotic stress response (to cold, salt, water deprivation, heat and oxidative stress) and phytohormone signaling (ABA, jasmonic acid, salicylic acid, auxin, ethylene and brassinosteroid) (Fig. 8). We also found that *MYB*, *NAC*, *bZIP* and *WRKY* transcription factor genes were up-regulated by more than twofold in the transgenic alfalfa (Table S2), and these regulatory genes may also play a key role in drought tolerance. In addition, the expression levels of a gene encoding PSII light-harvesting chlorophyll A/B-binding protein complex I (LHCB1, Medtr6g012110), and a gene encoding plastocyanin-like domain protein (Medtr6g023760) in photosystem were significantly increased in the transgenic lines by more than 2-fold. Genes involved in oxidative stress and redox regulation, including glutathione S-transferase (*GST*, Medtr7g065600), *laccase* (Medtr5g020600) and gibberellic acid-stimulated *Arabidopsis* gene family (*GASA*, Medtr7g090590), were elevated at various level in the transgenic lines (Fig. 8).

To further validate the RNA-seq data, we performed qRT-PCR on eight representative genes, belonging to WRKY, bZIP, MYB, NAC, GST and LHCB families, by using leaf samples that were different from those used RNA-seq. qRT-PCR results were highly consistent with those obtained by RNA-seq (Table S3), indicating that these genes were essentially affected by *γ-TMT* over-expression.

## Discussion

*γ-TMT* gene has been characterized in many plant species including *Arabidopsis* [30], *Brassica juncea* [31], tobacco [32] and soybean [33]. *γ-TMT* gene regulates α-tocopherol biosynthesis and plant physiological state in response to a variety of abiotic stresses [34, 35]. However, the function of *γ-TMT* gene was barely characterized in legume forage. In this study, we characterized *TMT* gene from alfalfa and found it is involved in the drought tolerance of transgenic alfalfa plants.

### *MsTMT* improves water use efficiency of alfalfa mainly by regulating stomatal development

We also found that over-expression of *MsTMT* promotes the metabolism of vitamin E and increased the accumulation of α-tocopherol (Fig. 2b). Most importantly, we found that *MsTMT* plays a role in drought tolerance that was supported by higher transpiration efficiency of transgenic plants.

The first step of photosynthesis is to capture the energy of light. LHCB1, a member of LHCII proteins, is the major pigment-binding protein and plays a role in absorbing sunlight and transferring the excitation energy to the reaction centers of photosystem [36, 37]. In this study, no difference in chlorophyll content was detected between control and transgenic plants under normal growth condition (Fig. 5a), but a significant increase in the expression level of *LHCB1* was detected (Table S2). Moreover, non-photochemical quenching (NPQ) increased in transgenic plants (Fig. S3). It means that excess energy absorbed in PSII antennae goes into thermal energy dissipation in the transgenic plants. A similar report also showed that increased α-tocopherol caused higher NPQ level that may be a positive role of α-tocopherol in keeping photosystems from oxidative damage [38]. Thus, our studies suggest that *MsTMT* regulates the photochemical energy of photosystem to a suitable level by balancing light absorption and NPQ.

Furthermore, we found that overexpressing *MsTMT* gene decreased the RuBP carboxylase activity and electron transfer rate (Fig. S3), which should be the main factors that caused slight reduction in CO_2_ assimilation in transgenic plants. Finally, decrease in both transpiration rate and stomatal conductance contributed to the higher instantaneous WUE in the transgenic alfalfa (Fig. 5b, d, e).

Stomatal pores are the primary conduits for CO_2_ and H_2_O exchange between plants and atmosphere on the surface of leaves, which play a critical role in regulating WUE. Many studies have shown that WUE can be promoted by direct genetic operation on stomatal number [11, 14, 39]. Stomatal development in *Arabidopsis* follows a stereotyped sequence of cell-fate transitions and cell divisions from the initial asymmetric cell division to the formation of mature stoma. The asymmetric cell division in establishing stomatal lineages has been extensively reviewed [40–43]. In this study, we found that the stomatal development on both sides of leaflets was dramatically inhibited in all transgenic lines (Fig. 6a-c). Epidermal phenotype analysis showed that this inhibition occurred before SLGCs stage and was similar to that of the phenotype in the *spch-1* mutants in *Arabidopsis*, which showed a block in stomatal development without formed stomata [26]. The expression level of *MsSPCH* in transgenic alfalfa was significantly reduced compared with control plants, indicates the possible involvement of *MsTMT* in modulating initial asymmetric cell division from protodermal cell to the MMC in stomatal development.

**Fig. 9:**
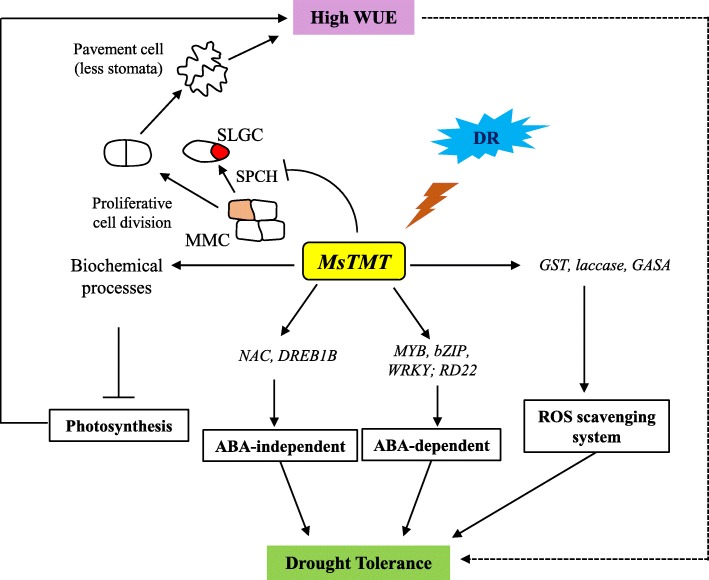
Simplified model of the molecular mechanism of *MsTMT* involved in plant water use efficiency and drought (DR) response. Overexpression of *MsTMT* in alfalfa represses the transcriptional level of *SPCH*, which plays a critical role in the initial asymmetric cell division of stomatal development from meristemoid mother cell (MMC) to stomatal-lineage ground cells (SLGCs). This repression causes more MMCs to differentiate into pavement cells resulting in reduced stomatal density that contributes to reduced transpiration rate and further to increased WUE, which probably in turn improves alfalfa drought tolerance. *MsTMT* also modulates photosynthesis by biochemical processes including light harvesting, non-photochemical quenching (NPQ), Rubisco carboxylation and electron transport capacity. However, the overlapping effect of these factors have limited effect on photosynthetic capacity. *MsTMT* may response to drought stress involved in ABA-dependent and -independent pathways by regulating *MYB*, *bZIP*, *WRKY*, and *RD22* and *NAC* and *DREB1B* genes, respectively. The increase in *MsTMT* gene expression was accompanied by elevated expression of glutathione S-transferase (*GST*), *laccase* and gibberellic acid-stimulated *Arabidopsis* (*GASA*) genes that play an important role in protection against oxidative stress

All these findings demonstrated that *MsTMT* gene affected plant transpiration efficiency primarily by coordinating stomatal development, via suppressing the expression of *MsSPCH* that regulates the formation of SLGCs (Fig. 9).

### *MsTMT* plays roles in stress response involved in multiple signaling pathways

Phytohormones are essential for plant growth and development, particularly in regulating plant adaptation to environmental stresses [44–47]. Among these phytohormones, ABA plays pivotal roles in mediating a wide range of plant responses to stresses, accompanied by a rapid accumulation when plants suffer from adverse conditions, which results in increased tolerance to abiotic stresses [45, 47]. In this study, overexpression of *MsTMT* improves plant performance under PEG-induced drought stress by alleviating oxidative damage to cell membrane (Fig. 4c-f) and osmotic substance (Fig. 4h). To validate whether this regulatory mechanism involved in ABA signaling, several marker genes involved ABA biosynthesis, ABA response and ABA signaling were detected by qRT-PCR [48–50]. Interestingly, the expression levels of *ABA1*, *ABA3* and *NCED3*, in ABA biosynthesis, were down regulated in transgenic lines compared with the control plants.

Furthermore, the transcriptional levels of typical genes that response to ABA (*RD22*, *DREB1B*) and those involved in ABA signaling (*ABI3/4*) were suppressed by the overexpression of *MsTMT* gene in alfalfa. This is consistent with our previous results that the expression of *MsTMT* was less sensitive to exogenous ABA treatment [51]. However, a conflicting result was reported in a previous study in the *Arabidopsis vte4* mutant, in which the *vte4* mutant and wild-type lines have similar ABA levels in rosettes under the normal condition, whereas a slight decrease occurred in *vte4* confronting salt stress compared with wild-type plants [35]. The variation in ABA response may indicate subtle functional differences of *TMT* gene from different species. It should be noted that many genes encoding transcriptional factors, such as WRKYs, MYBs and bZIP, involved in ABA signaling were significantly up-regulated in transgenic plants (Table S2), which suggested that overexpressing *MsTMT* gene partially restrained ABA signaling. Our analysis also showed *DREB1B* and other two NAC transcriptional factor genes were elevated in transgenic plants. Therefore, *MsTMT* play a key role in stress response through both ABA-dependent and ABA-independent pathways (Fig. 9).

Recent study showed that overexpression of *MsHPPD* gene negatively regulates ABA biosynthesis, which probably resulted from the consume of most geranylgeranyl diphosphate (GGDP) in the biosynthesis of tocopherol instead of in ABA biosynthesis [52]. Here, ABA content decreased almost 40% in the transgenic plants (Fig. 7h). Since *TMT* gene is positioned downstream of the tocopherol biosynthesis pathway, therefore whether or not tocopherol biosynthesis competing with ABA pathway needs to be verified in the future.

Extensive evidence indicates that JA, as crucial signaling molecular, is involved in many plant responses to abiotic stresses and probably interacts with ABA synthesis under water stress [53, 54]. Moreover, JA is related with the accumulation of antioxidants (including catalase, ascorbate peroxidase, GST and glutathione reductase). Exogenous JA application could greatly increase the antioxidant enzyme activities and their synthesis in plants [53, 55]. In our study, we noticed that overexpression of *MsTMT* gene gave rise to upregulations of a JA biosynthesis gene (Medtr5g023990) and *GST* gene (Medtr7g065600) respectively by 2.4- and 2.3-fold in transgenic alfalfa plants. In addition, expression levels of genes involved in GA were affected (1.7-fold increase and 1.8-fold decrease in GA20-oxidases) (Table S1, 2). Together, our results with previous reports strongly point that overexpression of *MsTMT* can directly or indirectly affect the biosynthesis of multiple phytohormones that may play a comprehensive role in stress tolerance.

## Conclusion

In this study, we discovered new functions of *TMT* gene in the regulation of WUE and drought tolerance in alfalfa. Overexpression of *MsTMT* improves plant WUE by repressing *MsSPCH* gene to inhibit stomatal development in alfalfa, and increasing tocopherol content. Additionally, *MsTMT* plays crucial roles in response to drought stress, which involves complex mechanisms including ROS scavenging capability, plant physiological development, and ABA-dependent or/and ABA-independent signaling pathways as illustrated in Fig. 9. Briefly, this study will contribute to the deep understanding on the role of *TMT* gene in higher plants and illustrated that *MsTMT* probably is a candidate gene for the improvement of plant drought tolerance by molecular breeding. Due to the effect of *MsTMT* on ROS scavenging and ABA biosynthesis, this study will also provide a theoretical basis for studies in other abiotic stresses.

## Methods

### Plant materials and PEG-simulated drought stress treatment

A commercial alfalfa (*Medicago saiva* L.) cultivar, Zhongmu No.1, was used in this study. The seeds of this cultivar were kindly provided by Prof. Qingchuan Yang (the Institute of Animal Science, Chinese Academy of Agricultural Sciences). Because alfalfa is an outcrossing species, the explants (leaves) used for transformation were vegetatively propagated from the same plant to ensure all transgenic plants have the same genetic background. Alfalfa was transformed as described by Fu et al. [56]. Both the transgenic and control (empty vector) alfalfa plants propagated from stem cuttings were grown in plastic culture pots filled with loam soil and perlite (3:1), under a photoperiod of 16 h/8 h and a temperature of 25 °C/20 °C for four weeks. Then, twelve alfalfa plants for each genotype were randomly assigned to well-watered or to stress treatment. Plants were subjected to drought treatment with 20% polyethylene glycol (PEG) 6000 for one week, followed by re-watering for 10 days for recovery.

### Determination of tocopherols

The fully expanded leaves were collected from three plants for each genotype and ground in liquid nitrogen. Approximately 50 mg leaf powder for each sample was extracted with methanol/chloroform (2:1, v/v) containing 0.01% butylated hydroxytoluene. After 20 min incubation at room temperature, 0.33 ml chloroform and 0.6 ml H_2_O were added into the homogenate independently. The mixture was centrifuged at 14,000 g for 10 min. Then, the organic phase was transferred to a clean triangular flask and dried in the rotary vacuum evaporator with water bath heating. The dry residue at the bottom was resolved in 0.4 ml dichloromethane/methanol (1:5, v/v) and analyzed at Institute of Botany, Chinese Academy of Sciences. Resolution of tocopherol species was achieved using ultra-high performance supercritical fluid chromatography–mass spectrometry (UHPSFC-MS) techniques. UHPSFC-MS analysis was carried out using a Waters Acquity UltraPerformance Convergence Chromatography (UPC2) system (Waters, Milford, MA, USA) coupled to a triple-Quadrupole Mass Spectrometry (XEVO-TQ) with electrospray ionization (ESI). Sample components were identified by mobility relative to individual tocopherol standards (Sigma-Aldrich, USA). Peak areas with those external standards were used for quantitation.

### qRT-PCR analysis

The fully expanded leaves under light were selected for gene expression analysis. Leaf samples were collected from three plants for each genotype. Total RNAs were extracted from alfalfa leaves using an Eastep Super Total RNA Extraction Kit (Promega, USA) following the indicated protocol. Then, cDNA was generated from 1 μg total RNA treated with RNase-free DNase I by SuperScript™ III reverse transcriptase (Invitrogen, USA). Finally, cDNA was diluted 10-fold and used as template in qRT-PCR with SYBR Green PCR Master Mix (Takara, Japan), on an ABI 7500 Real-Time PCR System (Bio-Rad, USA). Gene specific primers were designed according to Illumina EST reads and sequences of PCR-based cloning and were listed in Table S4. PCR cycling conditions consisted of one cycle at 95 °C for 30 s, and 40 cycles at 95 °C for 5 s, and 60 °C for 34 s, followed by one cycle of 95 °C for 15 s, 60 °C for 1 min, and 95 °C for 15 s. The relative quantitative results were calculated by the 2^-ΔΔ*C*^_T_ comparative method [57].

*MsActin* (EU664318) gene was used as the internal control in qRT-PCR analysis. qRT-PCR results were normalized by *MsActin* with three biological replicates.

### Leaf gas exchange measurements

Rates of CO_2_ assimilation (A), transpiration (E) and stomatal conductance to water vapour (g_w_) were measured with the LI-COR 6800 portable system (LI-COR Biosciences, USA). The first fully expanded leaf from the top of branch was selected for gas exchange measurement at a photosynthetic photon flux density (PPFD) of 1000 μmol m^− 2^ s^− 1^. Leaf temperature was set at the growth temperature (25 °C) and a relative humidity of 55%. Instantaneous leaf transpiration efficiency was calculated as the ratio of A to E.

To perform leaf area correction of gas exchange data, all leaves were immediately collected and scanned, and then used for stomata analysis. In this measurement, at least three plants were selected for each genotype and five leaves were detected for each plant.

### Carbon isotope analysis

Leaves at the same position as in gas exchange measurements were collected from three plants for each genotype and dried at 80 °C and ground into fine powder that passed through a 100-mesh screen. Leaves (2–3 mg) from one plant as a sample was weighed and used to analyze the carbon isotope composition at State Key Laboratory of Vegetation and Environmental Change, Chinese Academy of Sciences. The δ^13^C values were converted to carbon isotopic discrimination values (△^13^C) according to the equation [58]: △^13^C = (δa-δp)/(1 + δp), where δa and δp refer to the δ^13^C of atmospheric CO_2_ and plant material, respectively, and δa was assumed to be − 8 per mil.

### Stomatal density determination

The five fully expanded leaves from each plant used for gas exchange were selected to analyze the stomatal density. For each side of leaflet, we selected six fields of view symmetrically distributed on both sides of the vein for stomatal counting. The numbers of stomata and epidermal cells were counted on both sides of a leaflet at the middle part between margin and midrib. The stomata were examined and photographed with a light microscope (Leica DM5000, Germany) [59].

### Determination of stress-associated physiological indicators

For chlorophyll content analysis, leaf discs from transgenic and control plants were immersed in 80% acetone to extract chlorophyll [60]. Leaf temperature was estimated with a portable meter (model TYS-A; TOP Instrument, China).

To access leaf water status, mature leaves from transgenic and control plants were initially weighed. Subsequently, these leaves were left on filter paper in an illumination incubator and weighed again at 0.5, 1, 2, 3, 4, 5 and 6 h. Leaf water loss rate was calculated by the percentage of fresh weight loss to the initial leaf weight [61].

To detect the accumulation of superoxide and H_2_O_2_, the diaminobenzidine (DAB) staining was induced to determine the plant status under osmotic stress [62].

For membrane stability, electrolyte leakage was assessed under stress conditions [63]. Leaf samples were collected and washed three times with deionized water to remove surface-adhered electrolytes, and then transferred to a clean tube containing 10 ml of deionized water. The tube was incubated at room temperature on a shaker for 3 h, and the initial electrical conductivity (C_i_) was determined with a DDSJ2308A conductivity meter (Leisi, China). Then, the tubes with leaves were autoclaved for 20 min, and the final electrical conductivity (C_f_) was measured again. Electrolyte leakage was calculated by using the following formula: Relative electrolyte leakage (%) = (C_i_/C_f_) × 100.

A previous protocol was followed to measure malondialdehyde (MDA) content with some modifications [64]. Briefly, 0.5 mg fresh leaves were ground into a homogenate with quartz sand in 4 ml 10% (m/v) trichloroacetic acid (TCA) solution. The samples were then centrifuged at 4000 g for 10 min. Then, 2 ml supernatant was transferred into 2 ml 0.6% (m/v) thiobarbituric acid (TBA). Finally, the mixture was boiled for 15 min, and then quickly cooled and centrifuged again. The absorbance of the supernatant was measured at 450, 532, and 600 nm with a NanoPhotometer P330 (Implen, Germany). MDA content was estimated according to the formula: [6.45 × (A_532_-A_600_)]-0.56 × A_450_.

For the measurement of proline content, the samples were examined with a Pro Determination Kit (Cominbio, China). Fresh leaves of 0.1 g were ground in liquid nitrogen, and then 1 ml of extraction buffer were added, followed by water bath at 95 °C for 10 min. The mixture was centrifuged at 12,000 g for 10 min and the supernatant was used for proline analysis. The absorbance of the mixture was recorded at 520 nm with a NanoPhotometer P330 (Implen, Germany).

For the measurement of ABA content, the samples were detected as previous report [52] and performed at Institute of Genetics and Developmental Biology, Chinese Academy of Sciences.

The third to the fifth leaf from the top of branches were selected for the above measurements. At least three biological replicates were included for each genotype in each assay.

### RNA-seq analysis

Three biological replicates of the control and transgenic alfalfa (line Nos.16 and 17) leaf samples were used respectively for RNA-seq at Frasergen Bioinformatics Co., Ltd. (Wuhan, China). Briefly, total RNAs were extracted according to the instruction manual of the TRlzol Reagent (Life technologies, USA). mRNA was isolated by using NEBNext Poly(A) mRNA Magnetic Isolation Module (NEB, E7490, USA). The cDNA library was constructed following the manufacturer’s instructions with a NEBNext Ultra RNA Library Prep Kit for Illumina (NEB, E7530, USA) and NEBNext Multiplex Oligos for Illumina (NEB, E7500, USA). Then, the enriched mRNA was fragmented into approximately 250–300 bp RNA inserts, which were used to synthesize the first-strand cDNA and the second-strand cDNA. For the double-stranded cDNA, end-repair/dA-tail and adaptor ligation were performed. The suitable fragments were isolated by Agencourt AMPure XP beads (Beckman Coulter, Inc., USA), and enriched by PCR amplification. Finally, the libraries were sequenced using an Illumina HiSeqX sequencing platform. Low quality reads, such as adaptor, unknown nucleotides> 5%, or Q20 < 20% (percentage of sequences with sequencing error rates < 1%), were removed by a custom Perl script. The clean reads filtered from the raw reads were mapped to the reference genome of *Medicago truncatula*.

The gene abundance differences between the control and transgenic lines were calculated based on the ratio of the FPKM values (fragments per kilobase of exon per million fragments mapped). The false discovery rate (FDR) control method was used to identify the *P*-value in multiple tests to compute the significance of differences. Genes with an adjusted *P*-value < 0.01 were assigned as differentially expressed. Gene Ontology (GO) enrichment analysis of differentially expressed genes (DEGs) was implemented by the TopGO R package version 2.24.0. Line Nos. 16 and 17 showed a similar result as compared with control plants, therefore, line No. 17 was selected to present the transcriptome data.

All RNA-seq data were deposited at the National Center for Biotechnology Information (NCBI) Sequence Read Archive (SRA) under the BioProject accession number SRP152318.

### Statistical analysis

Data were analyzed with one-way analysis of variance (ANOVA) and comparison of mean values were analyzed by OriginPro 8.5 software.

## Supplementary information

**Additional file 1: Figure S1.** Identification of transgenic alfalfa plants. A. The T-DNA region of recombinant vector pBI121-*35S::MsTMT* and the positions of primers for detecting transgene insertion. B. Genomic PCR of alfalfa lines transformed with *MsTMT* gene.

**Additional file 2: Figure S2.** Sequence information of recombinant vector pBI121-*35S::MsTMT*.

**Additional file 3: Figure S3.** γ + β-tocopherol content of transgenic lines and wild type in mature alfalfa leaves. Values are the mean ± SEM. Significant differences: *P ≤ 0.05, **P ≤ 0.01, ***P ≤ 0.001.

**Additional file 4: Figure S4.***MsTMT* affects biochemical processes of photosynthesis. A. Response of CO_2_ assimilation rate of the first fully expanded leaves from top of branches to intercellular CO_2_ concentration under saturating light for control and transgenic plants. The curves showed theoretical relationships according Farquhar et al. (1980). B. Maximum Rubisco carboxylation rates (*V*_cmax_) and electron transport capacity (*J*_max_) were estimated from *A-C*_i_ curves. C. Induction of non-photochemical quenching (NPQ) after 20 min of dark adaptation using handheld leaf fluorometer (FluorPen FP 100, Photon System Instruments, Czech Republic). Vertical bars denote SEM taken from 5 biological replicates.

**Additional file 5: Figure S5.** ABA content in transgenic and control alfalfa leaves. Leaf samples were from different batches of alfalfa plants with similar growth stage. Values are the mean ± SEM. Significant differences: *P ≤ 0.05, **P ≤ 0.01, ***P ≤ 0.001.

**Additional file 6: Figure S6.** Functional annotation of differentially expressed transcripts. A. Histogram of GO classifications. B. KEGG classification. a: cellular processes; b: environmental information processing; c: genetic information processing; d: metabolism; e: organismal systems. C. KEGG enrichment analysis. Here only positively enriched metabolic pathways were exhibited. The size of dots indicates the gene number involving in the pathway. The color of dots corresponds to different Rich Factor. The greater the Rich factor, the greater the degree of pathway enrichment. Q value is the corrected *p* value.

**Additional file 7: Table S1.** Differentially expressed genes (DEG) between control and transgenic lines.

**Additional file 8: Table S2.** Selected genes regulated in plants overexpressing *MsTMT* compared to control plants identified by RNA-seq.

**Additional file 9: Table S3.** qRT-PCR analysis of representative genes identified as differentially expressed in overexpressing *MsTMT* plants by RNA-seq.

**Additional file 10: Table S4.** Primers used in this study.

## Data Availability

The datasets supporting the conclusions of this study are included within the article and its additional files. The raw data of RNA-seq was available in NCBI (accession number: SRP152318).

## Abbreviations

ABA: Abscisic acid
AN3: ANGUSTIFOLIA3
A: CO_2_ assimilation
bHLH: Basic helix-loop-helix transcription factor
CaMV: Cauliflower mosaic virus
C_i_: Intercellular CO_2_ concentration
DAB: Diaminobenzidine
DEGs: Differentially expressed genes
DR: Drought
EL: Electrolyte leakage
E: Transpiration
GASA: Gibberellic acid-stimulated Arabidopsis
GGDP: Geranylgeranyl diphosphate
GO: Gene ontology
GST: Glutathione S-transferase
HGA: Homogentisate
HPPD: p-Hydroxyphenylpyruvate dioxygenase
JA: Jasmonic acid
*J*_max_: Electron transport capacity
LHCB1: Light-harvesting chlorophyll A/B-binding protein complex I
MDA: Malondialdehyde
MMC: Meristemoid mother cell
NCBI: National Center for Biotechnology Information
NPQ: Non-photochemical quenching
PPFD: Photosynthetic photon flux density
ROS: Reactive oxygen species
SLGCs: Stomatal-lineage ground cells
SPCH: SPCHLESS
SRA: Sequence Read Archive
TBA: Thiobarbituric acid
TCA: Trichloroacetic acid
TMT: Tocopherol methyltransferase
*V*_cmax_: The maximum rate of Rubisco carboxylation
WUE: Water use efficiency
△: Carbon isotope discrimination

## References

[1] Lesk C, Rowhani P, Ramankutty N (2016). Influence of extreme weather disasters on global crop production. Nature..

[2] Asada K (2006). Production and scavenging of reactive oxygen species in chloroplasts and their functions. Plant Physiol.

[3] Sharma Pallavi, Jha Ambuj Bhushan, Dubey Rama Shanker, Pessarakli Mohammad (2012). Reactive Oxygen Species, Oxidative Damage, and Antioxidative Defense Mechanism in Plants under Stressful Conditions. Journal of Botany.

[4] Bartosz G (1997). Oxidative stress in plants. Acta Physiol Plant.

[5] Krishnamurthy Aparna, Rathinasabapathi Bala (2013). Oxidative stress tolerance in plants. Plant Signaling & Behavior.

[6] Szarka A, Tomasskovics B, Bánhegyi G (2012). The ascorbate-glutathione-α-tocopherol triad in abiotic stress response. Int J Mol Sci.

[7] Hetherington AM, Woodward FI (2003). The role of stomata in sensing and driving environmental change. Nature..

[8] Chaerle L, Saibo N, Van Der Straeten D (2005). Tuning the pores: towards engineering plants for improved water use efficiency. Trends Biotechnol.

[9] Yoo CY, Pence HE, Hasegawa PM, Mickelbart MV (2009). Regulation of transpiration to improve crop water use. Crit Rev Plant Sci.

[10] Casson SA, Hetherington AM (2010). Environmental regulation of stomatal development. Curr Opin Plant Biol.

[11] Franks PJ, Doheny-Adams TW, Britton-Harper ZJ, Gray JE (2015). Increasing water-use efficiency directly through genetic manipulation of stomatal density. New Phytol.

[12] Wang C, Liu S, Dong Y, Zhao Y, Geng A, Xia X, Yin W (2016). *PdEPF1* regulates water-use efficiency and drought tolerance by modulating stomatal density in poplar. Plant Biotechnol J.

[13] Hughes J, Hepworth C, Dutton C, Dunn JA, Hunt L, Stephens J, Waugh R, Cameron DD, Gray JE (2017). Reducing stomatal density in barley improves drought tolerance without impacting on yield. Plant Physiol.

[14] Meng LS, Yao SQ (2015). Transcription co-activator *Arabidopsis* ANGUSTIFOLIA3 (AN3) regulates water-use efficiency and drought tolerance by modulating stomatal density and improving root architecture by the transrepression of *YODA (YDA)*. Plant Biotechnol J.

[15] Liu Y, Yuan J, Ma H, Song J, Wang L, Weng Q (2015). Characterization and functional analysis of a B3 domain factor from *zea mays*. J Appl Genet.

[16] Munné-Bosch S, Alegre L (2002). The function of tocopherols and tocotrienols in plants. Crit Rev Plant Sci.

[17] Foyer CH, Shigeoka S (2011). Understanding oxidative stress and antioxidant functions to enhance photosynthesis. Plant Physiol.

[18] Čamagajevac ŠI, Pfeiffer ŽT, Maronić ŠD, Gupta D, Palma J, Corpas F (2018). Abiotic stress response in plants: the relevance of tocopherols. Antioxidants and antioxidant enzymes in higher plants.

[19] Evans HM, Bishop KS (1922). On the existence of a hitherto unrecognized dietary factor essential for reproduction. Science..

[20] Schneider C (2005). Chemistry and biology of vitamin E. Mol Nutr Food Res.

[21] DellaPenna D (2005). A decade of progress in understanding vitamin E synthesis in plants. J Plant Physiol.

[22] Espinoza A, Martín AS, López-Climent M, Ruiz-Lara S, Gómez-Cadenas A, Casaretto JA (2013). Engineered drought-induced biosynthesis of α-tocopherol alleviates stress-induced leaf damage in tobacco. J Plant Physiol.

[23] Khalatbari AA, Jaafar HZE, Khalatbari AM, Mahmood M, Othman R (2015). Wild type and *vte4* mutant *Arabidopsis thaliana* responses to different water frequencies: genetic engineering towards stress tolerance. J Soil Sci Plant Nutr.

[24] Jiang J, Jia H, Feng G, Wang Z, Li J, Gao H, Wang X (2016). Overexpression of *Medicago sativa TMT* elevates the α-tocopherol content in *Arabidopsis* seeds, alfalfa leaves, and delays dark-induced leaf senescence. Plant Sci.

[25] Yusuf MA (2010). Kumar D, Rajwanshi R, Strasser RJ, Tsimilli-Michael M, Govindjee, Sarin NB. Overexpression of γ-tocopherol methyl transferase gene in transgenic *Brassica juncea* plants alleviates abiotic stress: physiological and chlorophyll a fluorescence measurements. BBA-Bioenergetics..

[26] MacAlister CA, Ohashi-Ito K, Bergmann DC (2007). Transcription factor control of asymmetric cell divisions that establish the stomatal lineage. Nature..

[27] Torii KU (2012). Mix-and-match: ligand–receptor pairs in stomatal development and beyond. Trends Plant Sci.

[28] Shinozaki K, Yamaguchi-Shinozaki K (2000). Molecular responses to dehydration and low temperature: differences and cross-talk between two stress signaling pathways. Curr Opin Plant Biol.

[29] Finkelstein RR, Gampala SSL, Rock CD (2002). Abscisic acid signaling in seeds and seedlings. Plant Cell.

[30] Shintani D, DellaPenna D (1998). Elevating the vitamin E content of plants through metabolic engineering. Science..

[31] Yusuf MA, Sarin NB (2007). Antioxidant value addition in human diets: genetic transformation of *Brassica juncea* with *γ-TMT* gene for increased α-tocopherol content. Transgenic Res.

[32] Zhu Q, Zhang J, Yu H, Li L, Chen X, Jiang M, Tan M (2019). Maize cd-tolerant ZmVTE4 encoding γ-tocopherol-methyl-transferase alleviated cd-toxicity through its product α-tocopherol. Environ Exp Bot.

[33] Zhang L, Luo Y, Zhu Y, Zhang L, Zhang W, Chen R, Xu M, Fan Y, Wang L (2013). (2013). GmTMT2a from soybean elevates the α-tocopherol content in corn and *Arabidopsis*. Transgenic Res.

[34] Abbasi A, Hajirezaei M, Hofius D, Sonnewald U, Voll LM (2007). Specific roles of α- and γ-tocopherol in abiotic stress responses of transgenic tobacco. Plant Physiol.

[35] Ellouzi Hasna, Hamed Karim Ben, Cela Jana, Müller Maren, Abdelly Chedly, Munné-Bosch Sergi (2013). Increased sensitivity to salt stress in tocopherol-deficient Arabidopsis mutants growing in a hydroponic system. Plant Signaling & Behavior.

[36] Horie Y, Ito H, Kusaba M, Tanaka R, Tanaka A (2009). Participation of chlorophyll *b* reductase in the initial step of the degradation of light-harvesting chlorophyll *a*/*b*-protein complexes in *Arabidopsis*. J Biol Chem.

[37] Ruban Alexander V., Johnson Matthew P., Duffy Christopher D.P. (2012). The photoprotective molecular switch in the photosystem II antenna. Biochimica et Biophysica Acta (BBA) - Bioenergetics.

[38] Tang Yueli, Fu Xueqing, Shen Qian, Tang Kexuan (2016). Roles of MPBQ-MT in Promoting α/γ-Tocopherol Production and Photosynthesis under High Light in Lettuce. PLOS ONE.

[39] Masle J, Gilmore SR, Farquhar GD (2005). The *ERECTA* gene regulates plant transpiration efficiency in *Arabidopsis*. Nature..

[40] Nadeau JA, Sack FD, Somerville CR, Meyerowitz EM (2002). Stomatal development in *Arabidopsis*. The Arabidopsis book.

[41] Bergmann DC, Sack FD (2007). Stomatal development. Annu Rev Plant Biol.

[42] Pillitteri LJ, Torii KU (2012). Mechanisms of stomatal development. Annu Rev Plant Biol.

[43] Shpak ED (2013). Diverse roles of ERECTA family genes in plant development. J Integr Plant Biol.

[44] Santner A, Estelle M (2009). Recent advances and emerging trends in plant hormone signalling. Nature..

[45] Agarwal PK, Jha B (2010). Transcription factors in plants and ABA dependent and independent abiotic stress signaling. Biol Plantarum.

[46] Peleg Z, Blumwald E (2011). Hormone balance and abiotic stress tolerance in crop plants. Curr Opin Plant Biol.

[47] Yoshida T, Mogami J, Yamaguchi-Shinozaki K (2014). ABA-dependent and ABA-independent signaling in response to osmotic stress in plants. Curr Opin Plant Biol.

[48] Fujita Y, Fujita M, Satoh R, Maruyama K, Parvez MM, Seki M, Hiratsu K, Ohme-Takagi M, Shinozaki K, Yamaguchi-Shinozaki K (2005). AREB1 is a transcription activator of novel ABRE-dependent ABA signaling that enhances drought stress tolerance in *Arabidopsis*. Plant Cell.

[49] Yu Q, An L, Li W (2014). The CBL–CIPK network mediates different signaling pathways in plants. Palnt Cell Rep.

[50] Zhou Cheng, Ma Zhongyou, Zhu Lin, Xiao Xin, Xie Yue, Zhu Jian, Wang Jianfei (2016). Rhizobacterial Strain Bacillus megaterium BOFC15 Induces Cellular Polyamine Changes that Improve Plant Growth and Drought Resistance. International Journal of Molecular Sciences.

[51] Jia H, Wang X, Gao H, Dong J, Wang Y, Liu J, Shi Y (2012). Cloning the gene of γ-tocopherol methyltransferase (γ-TMT) from alfalfa and expression analysis in adverse situations. Acta Pratacul Sin.

[52] Jiang J, Chen Z, Ban L, Wu Y, Huang J, Chu J, Fang S, Wang Z, Gao H, Wang X. *P-hydroxyphenylpyruvate dioxygenase* from *medicago sativa* is involved in vitamin E biosynthesis and abscisic acid-mediated seed germination. Sci Rep. 2017. 10.1038/srep40625. 10.1038/srep40625 PMC5233959 28084442

[53] Brossa R, López-Carbonell M, Jubany-Marí T, Alegre L (2011). Interplay between abscisic acid and jasmonic acid and its role in water-oxidative stress in wild-type, ABA-deficient, JA-deficient, and ascorbate-deficient *Arabidopsis* plants. J Plant Growth Regul.

[54] Ismail A, Seo M, Takebayashi Y, Kamiya Y, Nick P (2015). A balanced JA/ABA status may correlate with adaptation to osmotic stress in *Vitis* cells. J Plant Physiol.

[55] Kaya A, Doganlar ZB (2016). Exogenous jasmonic acid induces stress tolerance in tobacco (*Nicotiana tabacum*) exposed to imazapic. Ecotox Environ Safe.

[56] Fu CX, Hernandez T, Zhou CE, Wang ZY, Wang K (2015). Alfalfa (*Medicago sativa* L.). Agrobacterium Protocols. Methods in Molecular Biology.

[57] Livak KJ, Schmittgen TD (2001). (2001). Analysis of relative gene expression data using real-time quantitative PCR and the 2^-ΔΔ*C*^_T_ method. Methods..

[58] Farquhar GD, Ehleringer JR, Hubick KT (1989). Carbon isotope discrimination and photosynthesis. Annu Rev Plant Physiol Mol Biol.

[59] Xie C, Zhang R, Qu Y, Miao Z, Zhang Y, Shen X, Wang T, Dong J (2012). Overexpression of *MtCAS31* enhances drought tolerance in transgenic *Arabidopsis* by reducing stomatal density. New Phytol.

[60] Ben Romdhane Walid, Ben-Saad Rania, Meynard Donaldo, Verdeil Jean-Luc, Azaza Jalel, Zouari Nabil, Fki Lotfi, Guiderdoni Emmanuel, Al-Doss Abdullah, Hassairi Afif (2017). Ectopic Expression of Aeluropus littoralis Plasma Membrane Protein Gene AlTMP1 Confers Abiotic Stress Tolerance in Transgenic Tobacco by Improving Water Status and Cation Homeostasis. International Journal of Molecular Sciences.

[61] Shi H, Chen L, Ye T, Liu X, Ding K, Chan Z (2014). Modulation of auxin content in *Arabidopsis* confers improved drought stress resistance. Plant Physiol Bioch.

[62] Xu J, Li XL, Luo L (2012). Effects of engineered *Sinorhizobium meliloti* on cytokinin synthesis and tolerance of alfalfa to extreme drought stress. Appl Environ Microbiol.

[63] Villar-Salvador P, Ocaña L, Peñuelas J, Carrasco I (1999). Effect of water stress conditioning on the water relations, root growth capacity, and the nitrogen and non-structural carbohydrate concentration of *Pinus halepensis* mill. (Aleppo pine) seedlings. Ann For Sci.

[64] Ma J, Yin C, Guo Q, Zhou M, Wang Z, Wu Y (2015). A novel DREB transcription factor from *Halimodendron halodendron* leads to enhance drought and salt tolerance in *Arabidopsis*. Biol Plantarum..

