# Supplementary figures and images for: Over-expression of a γ-tocopherol methyltransferase gene in vitamin E pathway confers PEG-simulated drought tolerance in alfalfa
Source: BMC Plant Biol. 2020 May 19;20:226. doi: 10.1186/s12870-020-02424-1 (PMC7238615; doi:10.1186/s12870-020-02424-1)

A

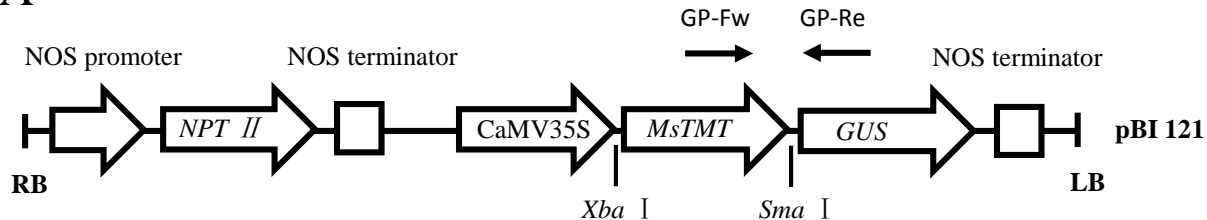

B

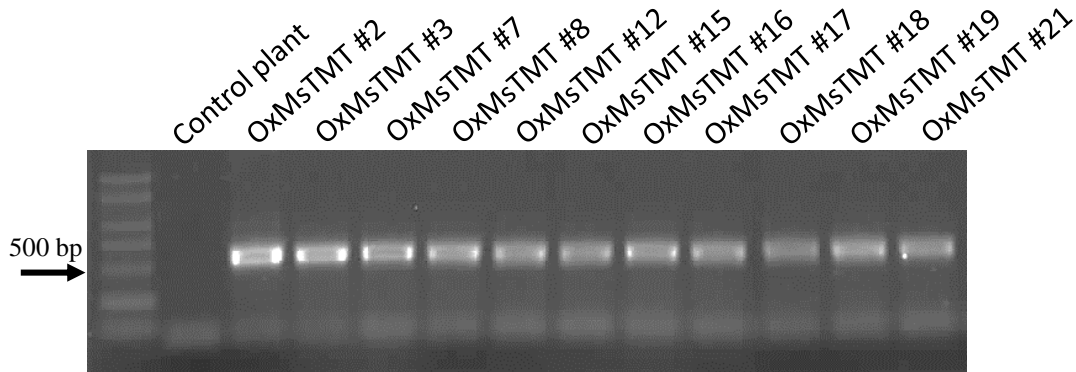

Supplement: Supplementary file 1 — Additional file 1: Figure S1. Identification of transgenic alfalfa plants. A. The T-DNA region of recombinant vector pBI121-35S::MsTMT and the positions of primers for detecting transgene insertion. B. Genomic PCR of alfalfa lines transformed with MsTMT gene. [file 12870_2020_2424_MOESM1_ESM.pdf]

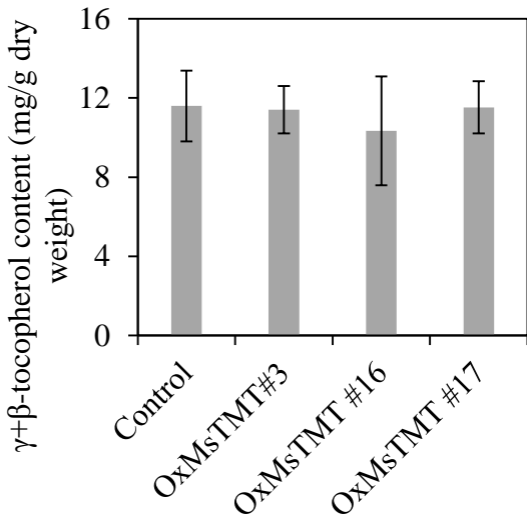

Supplement: Supplementary file 3 — Additional file 3: Figure S3. γ + β-tocopherol content of transgenic lines and wild type in mature alfalfa leaves. Values are the mean ± SEM. Significant differences: *P ≤ 0.05, **P ≤ 0.01, ***P ≤ 0.001. [file 12870_2020_2424_MOESM3_ESM.pdf]

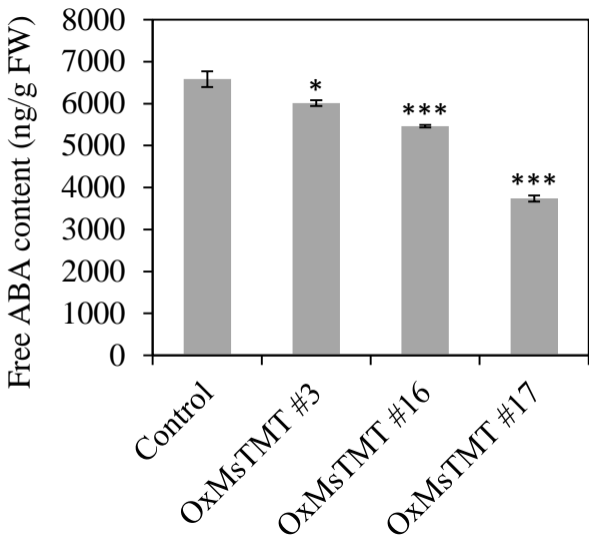

Supplement: Supplementary file 5 — Additional file 5: Figure S5. ABA content in transgenic and control alfalfa leaves. Leaf samples were from different batches of alfalfa plants with similar growth stage. Values are the mean ± SEM. Significant differences: *P ≤ 0.05, **P ≤ 0.01, ***P ≤ 0.001. [file 12870_2020_2424_MOESM5_ESM.pdf]

Fig. S6

A .

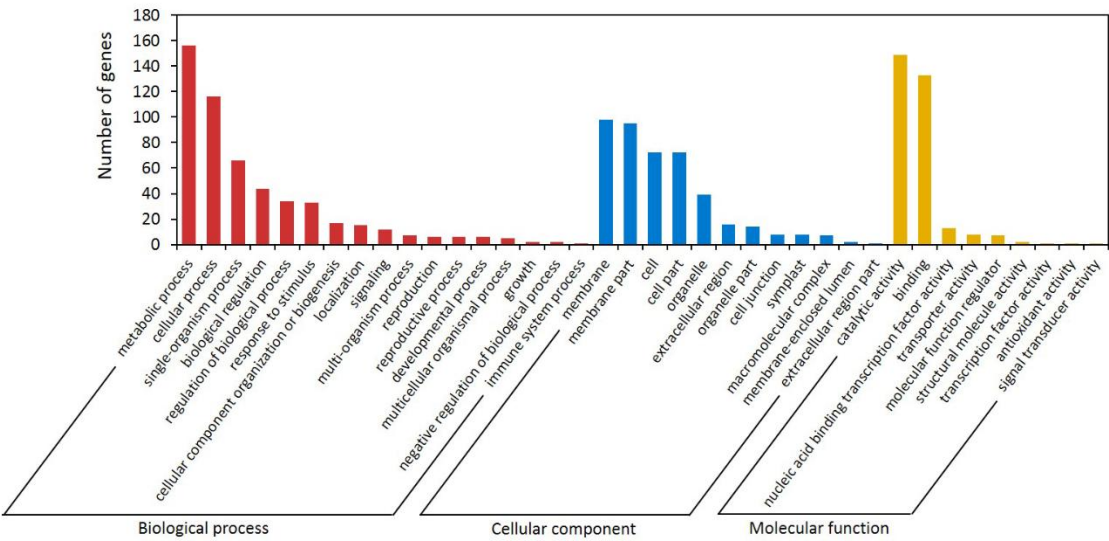

B .

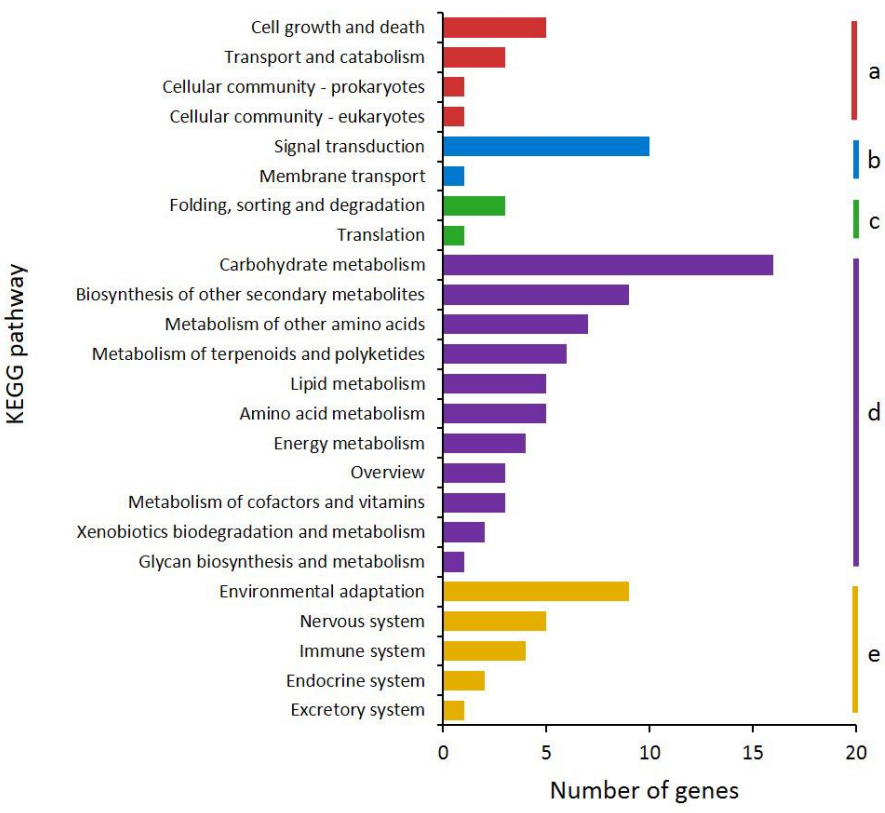

C .

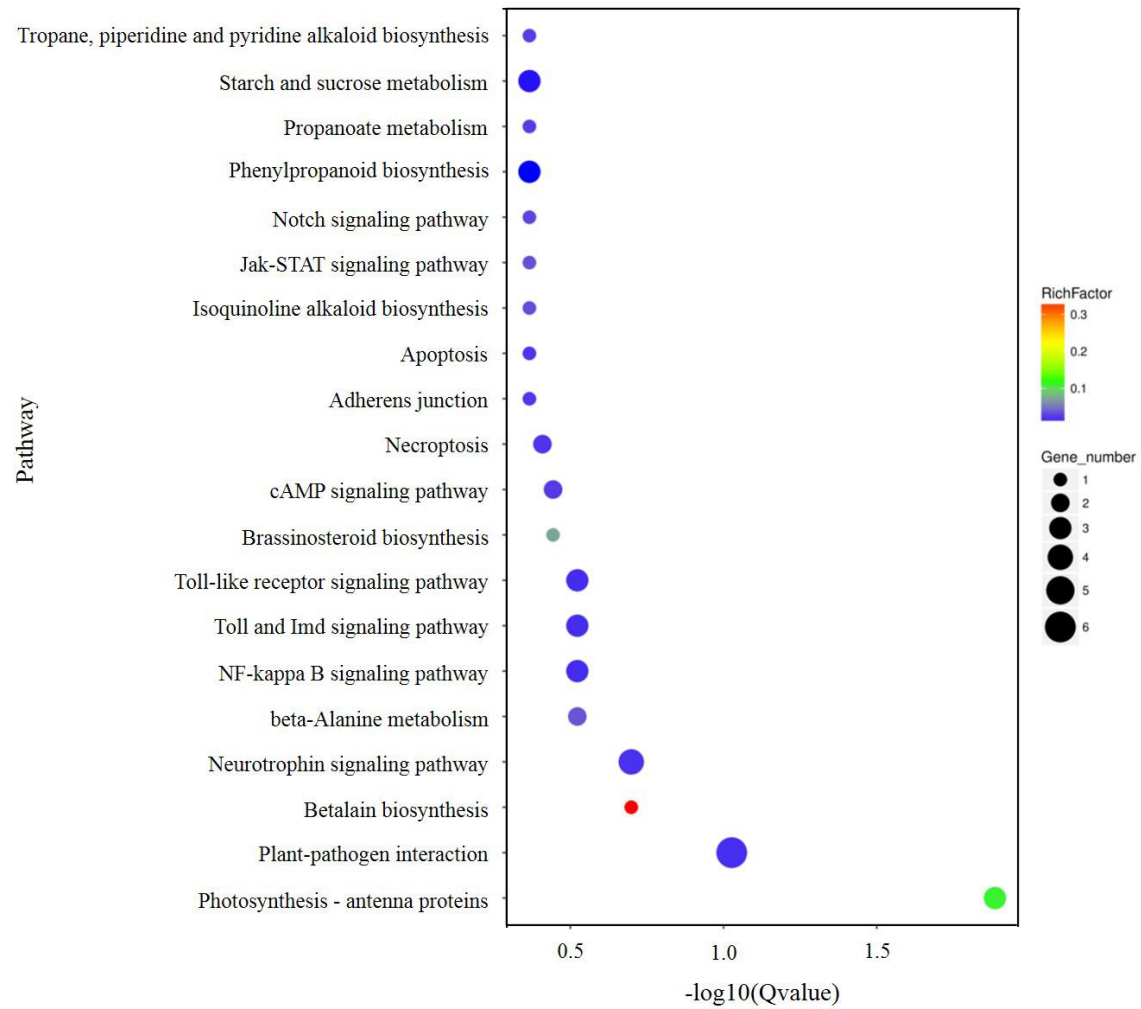

Supplement: Supplementary file 6 — Additional file 6: Figure S6. Functional annotation of differentially expressed transcripts. A. Histogram of GO classifications. B. KEGG classification. a: cellular processes; b: environmental information processing; c: genetic information processing; d: metabolism; e: organismal systems. C. KEGG enrichment analysis. Here only positively enriched metabolic pathways were exhibited. The size of dots indicates the gene number involving in the pathway. The color of dots corresponds to different Rich Factor. The greater the Rich factor, the greater the degree of pathway enrichment. Q value is the corrected p value. [file 12870_2020_2424_MOESM6_ESM.pdf]
