# Supplementary material for: Over-expression of a γ-tocopherol methyltransferase gene in vitamin E pathway confers PEG-simulated drought tolerance in alfalfa
Source: BMC Plant Biol. 2020 May 19;20:226. doi: 10.1186/s12870-020-02424-1 (PMC7238615; doi:10.1186/s12870-020-02424-1)
Supplement: Supplementary file 4 — Additional file 4: Figure S4.MsTMT affects biochemical processes of photosynthesis. A. Response of CO2 assimilation rate of the first fully expanded leaves from top of branches to intercellular CO2 concentration under saturating light for control and transgenic plants. The curves showed theoretical relationships according Farquhar et al. (1980). B. Maximum Rubisco carboxylation rates (Vcmax) and electron transport capacity (Jmax) were estimated from A-Ci curves. C. Induction of non-photochemical quenching (NPQ) after 20 min of dark adaptation using handheld leaf fluorometer (FluorPen FP 100, Photon System Instruments, Czech Republic). Vertical bars denote SEM taken from 5 biological replicates. [file 12870_2020_2424_MOESM4_ESM.pdf]

A

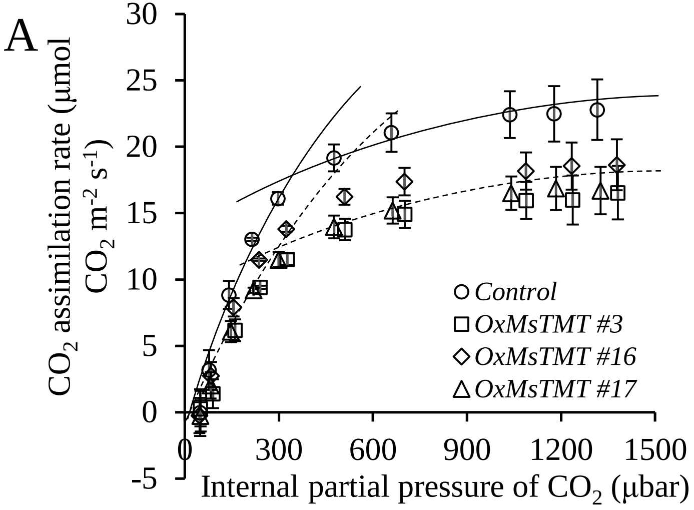

C

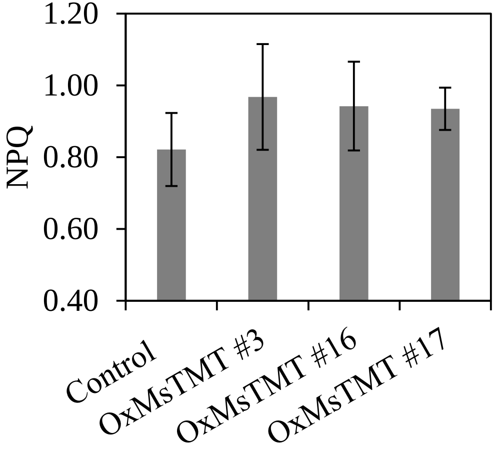

B

Genotype	V_{cmax} (μmol CO_2 m^{-2} s^{-1})	J_{max} (μmol e$^-$ m^{-2} s^{-1})
Control	33.84±3.63	31.10±3.76
OxMsTMT #3	26.74±1.90	22.49±2.42
OxMsTMT #16	26.38±2.41	22.65±1.36
OxMsTMT #17	25.67±3.84	23.15±4.17
