# Supplementary material for: Over-expression of a γ-tocopherol methyltransferase gene in vitamin E pathway confers PEG-simulated drought tolerance in alfalfa
Source: BMC Plant Biol. 2020 May 19;20:226. doi: 10.1186/s12870-020-02424-1 (PMC7238615; doi:10.1186/s12870-020-02424-1)
Supplement: Supplementary file 8 — Additional file 8: Table S2. Selected genes regulated in plants overexpressing MsTMT compared to control plants identified by RNA-seq. [file 12870_2020_2424_MOESM8_ESM.docx]

**Table S2.** Selected genes regulated in plants overexpressing *MsTMT* compared to control plants identified by RNA-seq. Significant expression difference was considered with a fold change ≥2.0-fold and FDR＜0.01.

| **Biological function** | | **GeneID** | **Fold change** | **Annotation** |
| --- | --- | --- | --- | --- |
|  | | Medtr1g101600 | -7.41 | CRT/DRE binding factor 4 |
|  | | Medtr4g086190 | -5.03 | AP2/ERF domain transcription factor |
|  | | Medtr6g465510 | -2.19 | dehydration-responsive element-binding protein |
|  | | Medtr6g465530 | -2.16 | dehydration-responsive element-binding protein |
|  | | Medtr4g086160 | -5.65 | AP2/ERF domain transcription factor |
| **Transcription and RNA binding** | | Medtr8g027345 | 9.99 | myb transcription factor |
|  |  | Medtr7g105170 | 4.20 | NAC transcription factor-like protein |
|  |  | Medtr1g015140 | 2.99 | WRKY family transcription factor |
|  |  | Medtr1g013760 | 3.77 | WRKY family transcription factor |
|  |  | Medtr3g104750 | 2.72 | WRKY family transcription factor |
|  |  | Medtr3g106060 | 2.74 | WRKY family transcription factor |
|  |  | Medtr8g099350 | -2.08 | WRKY family transcription factor |
|  |  | Medtr4g079500 | 3.78 | bZIP family transcription factor |
|  |  | Medtr7g102790 | 2.50 | PLATZ transcription factor family protein |
|  |  | Medtr2g437130 | 3.13 | transcription termination factor family protein |
|  |  | Medtr8g100075 | 2.58 | methyltransferase PMT16, putative |
| **Signal transduction and intracellular trafficking** | | Medtr3g088655 | 2.72 | EF hand calcium-binding family protein |
|  |  | Medtr6g083730 | 2.80 | calmodulin-binding-like protein |
|  |  | Medtr3g089070 | -6.03 | EF hand calcium-binding family protein |
|  |  | Medtr6g028030 | 3.25 | annexin D8 |
|  |  | Medtr3g062570 | 4.38 | LRR receptor-like kinase |
|  |  | Medtr5g082460 | 2.07 | LRR receptor-like kinase family protein |
|  |  | Medtr8g469780 | 2.68 | LRR receptor-like kinase family protein |
|  |  | Medtr7g446160 | 5.96 | LRR receptor-like kinase family protein |
|  |  | Medtr8g469570 | 3.05 | LRR receptor-like kinase family protein |
|  |  | Medtr4g029710 | 2.13 | LRR receptor-like kinase |
|  |  | Medtr0087s0070 | 2.70 | LRR receptor-like kinase |
|  |  | Medtr1g105750 | 3.80 | cysteine-rich receptor-kinase-like protein |
|  |  | Medtr1g105800 | 2.98 | cysteine-rich receptor-kinase-like protein |
|  |  | Medtr1g105885 | 4.28 | cysteine-rich RLK (receptor-like kinase) protein |
|  |  | Medtr6g083780 | 3.25 | receptor-like kinase |
|  |  | Medtr1g028170 | 2.27 | stress-induced receptor-like kinase |
|  |  | Medtr1g105615 | 2.94 | cysteine-rich receptor-kinase-like protein |
|  |  | Medtr1g105820 | 2.45 | cysteine-rich receptor-kinase-like protein |
|  |  | Medtr6g057750 | 2.40 | cysteine-rich receptor-kinase-like protein |
|  |  | Medtr4g114280 | 2.56 | lectin kinase family protein |
|  |  | Medtr1g105755 | 3.68 | cysteine-rich receptor-kinase-like protein |
|  |  | Medtr5g019050 | 2.84 | LysM-domain receptor-like kinase |
|  |  | Medtr3g011930 | 2.49 | cysteine-rich receptor-like kinase |
|  |  | Medtr1g027160 | 2.54 | stress-induced receptor-like kinase |
|  |  | Medtr3g067775 | 2.93 | tyrosine kinase family protein |
|  |  | Medtr6g016200 | 2.89 | leucine-rich receptor-like kinase family protein |
|  |  | Medtr2g096970 | 2.00 | kinase 1B |
|  |  | Medtr5g023980 | -2.44 | Serine/Threonine-kinase Cx32 |
|  |  | Medtr7g053200 | -3.43 | Serine/Threonine-kinase OXI1-like protein |
|  |  | Medtr1g088965 | -3.34 | kinase interacting (KIP1-like) family protein |
| **Cellular processes** | | Medtr7g021950 | 3.51 | cyclin-like F-box protein |
| **Antioxidant and redox regulation** | | Medtr7g065600 | 2.30 | glutathione S-transferase |
|  |  | Medtr5g020600 | 2.03 | laccase/diphenol oxidase family protein |
|  |  | Medtr7g090590 | 2.11 | GASA/GAST/Snakin |
| **Photosynthesis** | | Medtr6g012110 | 2.22 | light-harvesting complex I chlorophyll A/B-binding protein |
|  |  | Medtr6g023760 | 2.14 | plastocyanin-like domain protein |
| **Ion transport** | | Medtr3g093020 | 4.78 | heavy metal transport/detoxification superfamily protein |
|  |  | Medtr4g057765 | 2.50 | heavy metal transport/detoxification superfamily protein |
|  |  | Medtr3g463730 | 2.21 | ammonium transporter 1 protein |
|  |  | Medtr6g034975 | 2.54 | vacuolar iron transporter-like protein |
| **Metabolism** |  | Medtr8g074335 | 3.46 | Chitinase (Class Ib) |
|  |  | Medtr6g023340 | 2.19 | chitinase |
|  |  | Medtr1g015970 | 3.83 | fructan exohydrolase |
|  |  | Medtr8g065010 | 3.36 | polygalacturonase plant-like protein |
|  |  | Medtr3g083580 | 2.83 | O-glycosyl hydrolase family 17 protein |
|  | **Carbohydrate** | Medtr3g111410 | 2.75 | rhamnogalacturonate lyase B-like protein |
|  |  | Medtr3g463370 | 2.71 | legume lectin beta domain protein |
|  |  | Medtr5g034090 | 2.13 | polygalacturonase |
|  |  | Medtr8g065030 | 2.01 | pectin lyase superfamily protein |
|  |  | Medtr7g050425 | 3.24 | phosphoglycerate mutase family protein |
|  |  | Medtr3g064610 | 3.70 | sucrose synthase |
|  |  | Medtr2g015850 | 2.87 | papain family cysteine protease |
|  |  | Medtr4g080360 | 2.34 | papain family cysteine protease |
|  |  | Medtr3g116080 | 1.64 | papain family cysteine protease |
|  |  | Medtr8g063060 | 3.89 | M20/M25/M40 family peptidase |
|  | **Amino acids and Proteins** | Medtr6g044810 | 3.06 | Kunitz type trypsin inhibitor / Alpha-fucosidase |
|  |  | Medtr1g006990 | 2.87 | subtilisin-like serine endopeptidase family protein |
|  |  | Medtr1g007030 | 2.59 | subtilisin-like serine endopeptidase family protein |
|  |  | Medtr8g033150 | 2.30 | subtilisin-like serine protease |
|  |  | Medtr2g089755 | 2.75 | HXXXD-type acyl-transferase family protein |
|  | **Lipids** | Medtr5g023730 | 5.31 | fatty acyl-CoA synthetase family protein |
|  |  | Medtr5g094210 | 2.65 | Lipid transfer protein |
|  |  | Medtr4g058820 | 2.39 | enoyl-(acyl carrier) reductase |
|  |  | Medtr4g058840 | 2.39 | enoyl-(acyl carrier) reductase |
|  |  | Medtr4g091440 | 3.70 | acyl-coenzyme A oxidase |
|  | **DNA/RNA modification** | Medtr1g102410 | 2.70 | DEAD-box ATP-dependent RNA helicase |
|  |  | Medtr2g076970 | 3.86 | P-loop nucleoside triphosphate hydrolase superfamily protein |
|  |  | Medtr6g009520 | 3.07 | P-loop nucleoside triphosphate hydrolase superfamily protein |
|  |  | Medtr3g064190 | 2.85 | P-loop nucleoside triphosphate hydrolase superfamily protein |
|  |  | Medtr1g019990 | 2.88 | P-loop nucleoside triphosphate hydrolase superfamily protein |
|  |  | Medtr6g009540 | 2.26 | P-loop nucleoside triphosphate hydrolase superfamily protein |
|  |  | Medtr1g492760 | 2.79 | HhH-GPD base excision DNA repair family protein |
|  |  | Medtr4g094658 | 3.37 | pentatricopeptide (PPR) repeat protein |
|  |  | Medtr8g036260 | 8.47 | suppressor-of-white-APricot splicing regulator |
|  | **Others** | Medtr2g089650 | 4.00 | anthocyanin 5-aromatic acyltransferase |
|  |  | Medtr1g037370 | 2.92 | cytochrome P450 family protein |
| **Phytohormone biosynthesis** | **jasmonic acid** | Medtr5g023990 | 2.40 | lipoxygenase |
|  | [**cytokinin**](file:///C:\Users\ll\AppData\Local\youdao\dict\Application\7.0.1.0214\resultui\dict\result.html?keyword=cytokinin) | Medtr4g117330 | 2.87 | adenylate isopentenyltransferase |
