# Supplementary material for: Over-expression of a γ-tocopherol methyltransferase gene in vitamin E pathway confers PEG-simulated drought tolerance in alfalfa
Source: BMC Plant Biol. 2020 May 19;20:226. doi: 10.1186/s12870-020-02424-1 (PMC7238615; doi:10.1186/s12870-020-02424-1)
Supplement: Supplementary file 9 — Additional file 9: Table S3. qRT-PCR analysis of representative genes identified as differentially expressed in overexpressing MsTMT plants by RNA-seq. [file 12870_2020_2424_MOESM9_ESM.docx]

Table S3. qRT-PCR analysis of representative genes identified as differentially expressed in overexpressing *MsTMT* plants by RNA-seq.

| Gene name | Gene ID | Digital expression (log2FC) | qRT-PCR |
| --- | --- | --- | --- |
| *WRKY* | Medtr1g013760 | 3.77 | 2.95 |
|  | Medtr1g015140 | 2.99 | 2.05 |
|  | Medtr3g106060 | 2.74 | 1.80 |
| *bZIP* | Medtr4g079500 | 3.78 | 5.67 |
| *MYB* | Medtr8g027345 | 9.99 | 13.25 |
| *NAC* | Medtr7g105170 | 4.20 | 1.36 |
| *GST* | Medtr7g065600 | 2.30 | 2.04 |
| *LHCB1* | Medtr6g012110 | 2.22 | 2.27 |

log2FC: log2 fold change for differentially expressed genes.
