# Supplementary material for: Over-expression of a γ-tocopherol methyltransferase gene in vitamin E pathway confers PEG-simulated drought tolerance in alfalfa
Source: BMC Plant Biol. 2020 May 19;20:226. doi: 10.1186/s12870-020-02424-1 (PMC7238615; doi:10.1186/s12870-020-02424-1)
Supplement: Supplementary file 10 — Additional file 10: Table S4. Primers used in this study. [file 12870_2020_2424_MOESM10_ESM.docx]

Table S4. Primers used in this study.

| **Aim or Gene name** | **Primer sequence** |
| --- | --- |
| Genotyping | GP-Fw: GGACATCAAATCAGCAGATTGGTC |
|  | GP-Re: CACACTTTGCCGTAATGAGTGACC |
| *MsActin* (EU664318) | Fw: GAGCGTTTCCGTTGTCCTGA |
|  | Re: AGGTGCTGAGGGAAGCCAAA |
| *MsSPCHLESS* (*SPCH*) | Fw: AAGAGACAGAAGATTGTAGAGGAACAA |
|  | Re: ACTTTCAACTATTGATGCTTGATCTCC |
| *MsRD22* | Fw: GGAATCTATGGTTGATTTCACTACTTCG |
|  | Re: CTGCATAAGGGTAATTCTCTTTGTGAC |
| *MsDREB1B* | Fw: GACTACTGACATTGACACGGTTGTG |
|  | Re: CTCTGGCATATTCAACACTTCCTCTTC |
| *MsNCED3* | Fw: GTCGCCTGTTGTTTACGATAAGG |
|  | Re: CAGAAACACTCTGGTGCATCAATC |
| *MsABA1* | Fw: CAGTGGTGTTGGATAATGGTCAGAAATAT |
|  | Re: GCCAGAGTATGTAGCTTCTGTTGCC |
| *MsABA3* | Fw: GTTGTTCGTAGAGTCTCCACATTGC |
|  | Re: CAGAATATCGTAACAAAGTACAAGGTCTTC |
| *MsABI3* | Fw: AACAACATCAACAAATTTCAATGATAGTG |
|  | Re: TTCCTCAATATTCACTTGTTGTGGTT |
| *MsABI4* | Fw: CGTGCTGCTATTATTCTCTATGGTTC |
|  | Re: GTAAACTGAGAAACCAGAAGGACGAG |
| *MsWRKY* | Fw: TCAATGGAGGAAATATGGTCAGAAG |
|  | Re: AGGAGCTTCTTCACTTTGGCTTG |
|  | Fw: CCTTGGATTAGGGCTGTTCTTAACC |
|  | Re: GAGTGGAAACGATAATTGGAAGAAGG |
|  | Fw: GTGCTCCTTTGCTCCTGGCTG |
|  | Re: ATGGTTGTGCTCTCCTTCGTATGTG |
| *MsbZIP* | Fw: TAGCCCAAGACAAAATCTTCAAAGAT |
|  | Re: GTGACACTGCTGCTGCTGTAGC |
| *MsMYB* | Fw: GAAGGAACACAACAAGTGACAAACAAC |
|  | Re: GACTCACATTCCATCTCTAACCTTTC |
| *MsNAC* | Fw: GATAGACGACCACCACCAAGGAG |
|  | Re: GCTCTCTGGTGAGTTTGTTGCAC |
| *MsGST* | Fw: CAGAATCCCTTGTGATTCTTGAGTATG |
|  | Re: TCTCTTCTGTTTCCTCAATAGCTTTCTC |
| *MsLHCB1* | Fw: GCGAGGGTGGACTTGACTACTTG |
|  | Re: AAGAGGTCCACCAGCAATACGG |
